# Supplementary material for: Multi-omics and machine learning reveal context-specific gene regulatory activities of PML::RARA in acute promyelocytic leukemia
Source: Nat Commun. 2023 Feb 9;14:724. doi: 10.1038/s41467-023-36262-0 (PMC9911410; doi:10.1038/s41467-023-36262-0)

Multi-omics and machine learning reveal context-specific gene regulatory activities of PML::RARA in Acute Promyelocytic Leukemia – Supplementary Figures

Villiers et al Figure S1

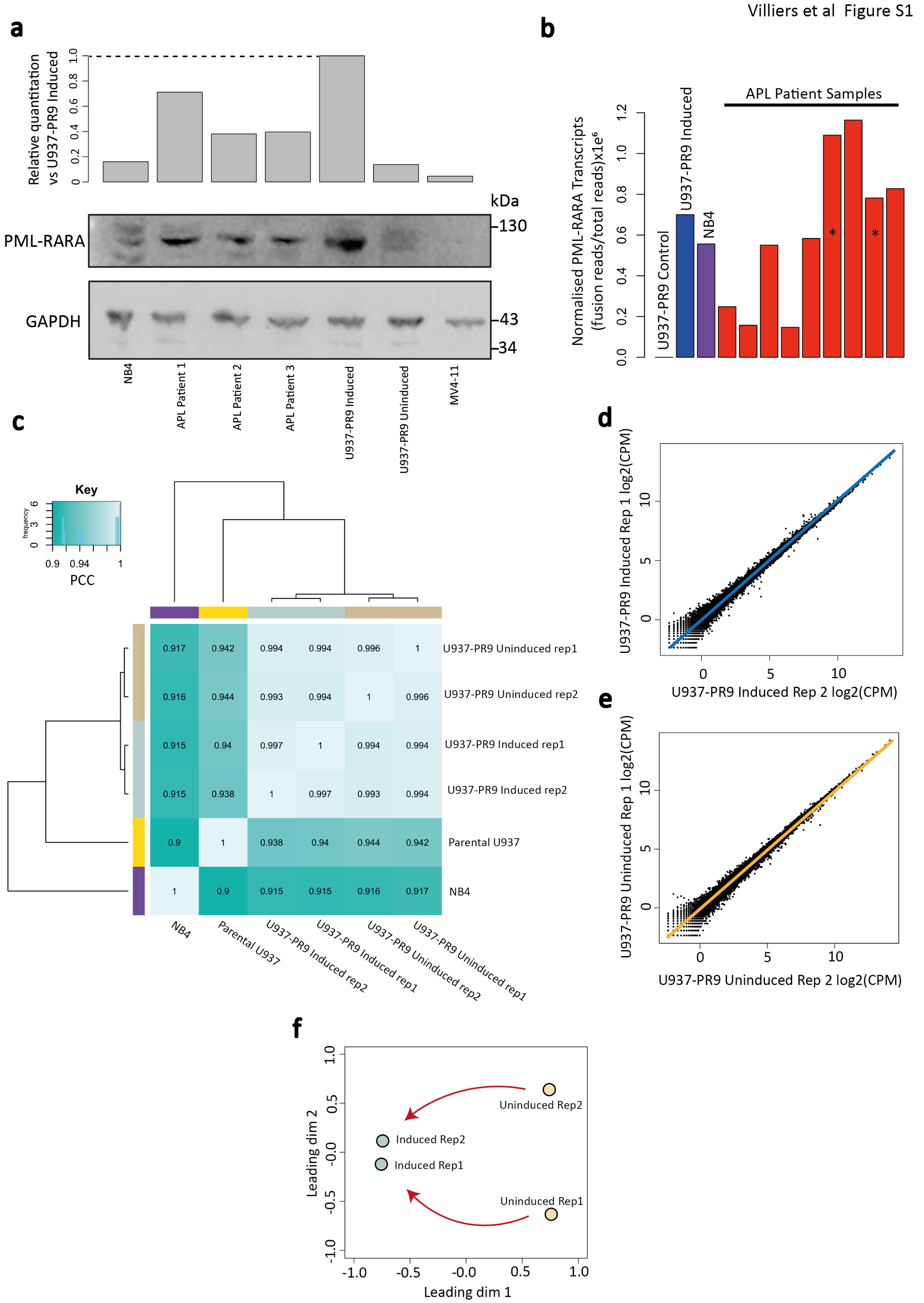

Supplementary Fig. 1: RNA-seq QC.

a Western blot showing the PML::RARA and GAPDH (loading control) protein expression levels in whole cell lysates from NB4, three APL patients, U937-PR9 induced, U937-PR9 uninduced and MV4-11 cells. The bar plot above the blots shows the semi-quantitative expression levels calculated from

each band's normalised intensities vs loading control (Methods). Each quantification is further normalised relative to the U937-PR9 induced expression level, with 1 representing a 100% expression level of the induced U937-PR9 band. **b** Barplots showing the normalised number of PML::RARA fusion transcripts detected using the Arriba Fusion detection tool (Methods). Briefly each RNA-seq dataset (U937-PR9 induced/uninduced, NB4, and nine in-house APL patients) was used as input into the Arriba tool and the number of reads that span both PML and RARA exons were counted, these were normalised by the number of total reads for that sample. * indicate the two patient samples used to generate capture Hi-C libraries. **c** Heatmap clustering the Pearson correlations of RNA-seq experiments. Correlations are based on correlating the CPM of each RNA-seq experiment. Each U937-PR9 uninduced and induced replicate is included, as well as publicly available NB4 and U937 RNA-seq data. Mean expression levels across four replicates from each publicly available dataset were used for comparison. PCC are included within each comparison and hierarchical clustering is applied based on PCC similarities. **d** Scatter plot showing the correlation of induced U937-PR9 cell replicates. Correlations are based on the counts per million of each RNA-seq experiment. **e** Scatter plot showing the correlation of induced U937-PR9 cell replicates. Correlations are based on the counts per million of each RNA-seq experiment. CPM = Counts Per Million, PCC = Pearson Correlation Coefficient. **f** Multidimensional scaling plot showing the similarity of each U937-PR9 replicate based on their gene expression profiles. Gold = uninduced, blue = induced. Arrows connect datasets from each experiment and emphasize that gene expression profiles after PML::RARA induction converge tightly. Source data are provided as a Source Data file – the corresponding data are also used in the main Figure 1.

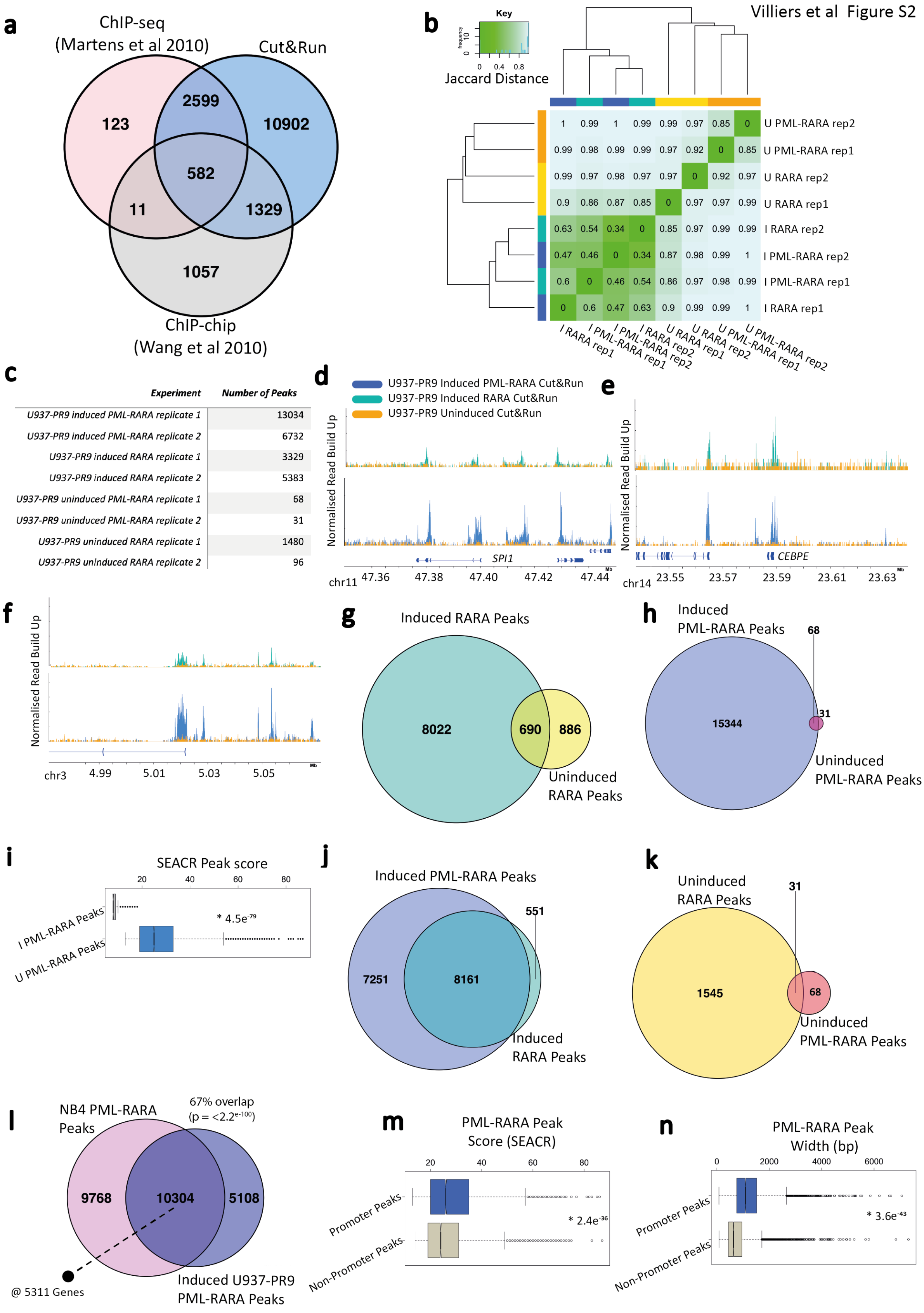

Villiers et al Figure S2
a
ChIP-seq
(Martens et al 2010)
Cut&Run
123
2599
10902
582
11
1329
1057
ChIP-chip
(Wang et al 2010)
b
Key
Jaccard Distance
U PML-RARA rep2
U PML-RARA rep1
U RARA rep2
U RARA rep1
I RARA rep2
I PML-RARA rep2
I PML-RARA rep1
I RARA rep1
c
Experiment | Number of Peaks
U937-PR9 induced PML-RARA replicate 1 | 13034
U937-PR9 induced PML-RARA replicate 2 | 6732
U937-PR9 induced RARA replicate 1 | 3329
U937-PR9 induced RARA replicate 2 | 5383
U937-PR9 uninduced PML-RARA replicate 1 | 68
U937-PR9 uninduced PML-RARA replicate 2 | 31
U937-PR9 uninduced RARA replicate 1 | 1480
U937-PR9 uninduced RARA replicate 2 | 96
d
U937-PR9 Induced PML-RARA Cut&Run
U937-PR9 Induced RARA Cut&Run
U937-PR9 Uninduced Cut&Run
Normalised Read Build Up
SPI1
chr11 47.36 47.38 47.40 47.42 47.44
e
Normalised Read Build Up
CEBPE
chr14 23.55 23.57 23.59 23.61 23.63
f
Normalised Read Build Up
chr3 4.99 5.01 5.03 5.05
g
Induced RARA Peaks
8022
690
886
Uninduced
RARA Peaks
h
Induced
PML-RARA Peaks
68
15344
31
Uninduced
PML-RARA Peaks
i
SEACR Peak score
20 40 60 80
I PML-RARA Peaks
U PML-RARA Peaks
* 4.5e-79
j
Induced PML-RARA Peaks
551
7251
8161
Induced
RARA Peaks
k
Uninduced
RARA Peaks
31
1545
68
Uninduced
PML-RARA Peaks
l
NB4 PML-RARA
Peaks
67% overlap
(p = <2.2e-100)
9768
10304
5108
@ 5311 Genes
Induced U937-PR9
PML-RARA Peaks
m
PML-RARA Peak
Score (SEACR)
20 40 60 80
Promoter Peaks
Non-Promoter Peaks
* 2.4e-36
n
PML-RARA Peak
Width (bp)
0 2000 4000 6000
Promoter Peaks
Non-Promoter Peaks
* 3.6e-43

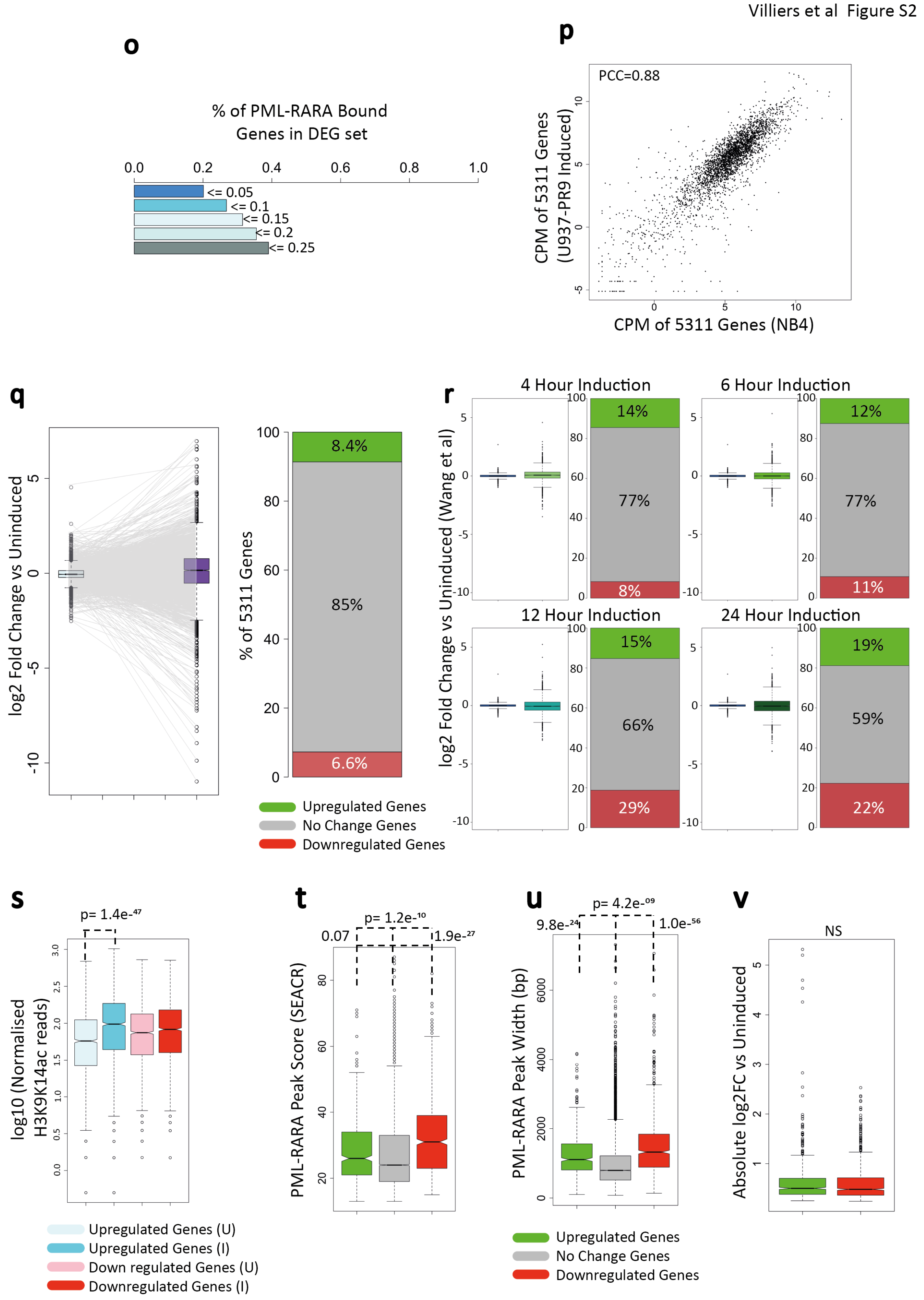

Supplementary Fig. 2: Cut&Run library QC.

a Venn diagram showing the overlap of PML::RARA peaks identified by Cut&Run in this study (blue) with peaks identified by Martens et al by ChIP-seq (pink) and by Wang et al by ChIP-ChIP (grey). **b**

Heatmap showing the Jaccard dissimilarity coefficient of each Cut&Run replicate peak set. The smaller the number the more similar the peak sets. **c** Table displaying total number of significant peaks detected in each replicate Cut&Run experiment. **d-f** Genomic tracks showing normalized read coverage in Cut&Run experiments at three key myeloid genes: *SPI1*, *CEBPE* and *BHLHE40*. Top track shows an experiment using an anti-RARA antibody in uninduced (orange) and induced (green) U937-PR9 cells. The bottom track shows an experiment using an anti-PML::RARA antibody in uninduced (orange) and induced (blue) U937-PR9 cells. Y axis represents the read count normalized by library size. **g** Venn diagram showing the overlap of all RARA peaks identified in induced (green) and uninduced (yellow) U937-PR9 cells. **h** Venn diagram showing the overlap of all PML::RARA peaks identified in induced (blue) and uninduced (pink) U937-PR9 cells. **i** Notched box plots showing the strength of PML::RARA binding at the 99 uninduced peaks (grey) and the 15,412 induced peaks (blue). * indicates the two-sided t-test p-value. N = 2 biological replicates of each Cut&Run condition. **j** Venn diagram showing the overlap of all PML::RARA peaks (blue) and all RARA peaks (green) in induced U937-PR9 cells. **k** Venn diagram showing the overlap of all PML::RARA peaks (pink) and all RARA peaks (yellow) in uninduced U937-PR9 cells. **l** Venn diagram showing the overlap of the 15,412 PML::RARA peaks in induced U937-PR9 cells from Cut&Run and PML::RARA peaks identified in NB4 cells from Cut&Tag. P indicates a significantly greater number of overlaps by chance, as determined by Chi-Square test. The total number of gene promoters (5,311) with a PML::RARA peak in both NB4 and U937-PR9 cells is shown. **m** Notched box plots showing the PML::RARA binding strength (based on the SEACR peak calling score) of peaks at gene promoters (blue) and peaks at non-promoters (grey). * indicates the two-sided t-test p-value. N = 2 biological replicates of each Cut&Run condition. **n** Notched box plots showing the width of PML::RARA peaks at gene promoters (blue), and non-promoters (grey). * indicates the two-sided t-test p-value. N = 2 biological replicates of each Cut&Run condition. **o** Bar plot showing the percentages of PML::RARA bound genes that are significantly differentially expressed at different significance thresholds. Benjamini-Hochberg adjusted p-value thresholds are displayed on the x-axis. DEG = Differentially Expressed Genes. **p** Correlation plot illustrating the expression levels of the 5,311 genes with PML::RARA binding in induced U937-PR9 cells (y-axis) and NB4 cells (x-axis). Expression levels refer to log2 CPM per gene. PCC =Pearson correlation coefficient. **q** Notched box plot (left) for 5,311 genes identified in Supplementary Fig. 2p, whose expression was unchanged upon U937-PR9 cell induction, comparing the induced U927-PR9 cells (light blue) and NB4 cells (purple) expression differences, relative to uninduced U937-PR9 cells. The grey lines connect the same genes in the two cell lines. The side bar plot (right) categorizes the percentage of genes whose fold difference is higher (green), equivalent (<0.5 log2 FC) (grey) or lower (red) in NB4 cells. **r** Notched box plots comparing the fold changes of the 4,656 PML::RARA-bound genes with no significant expression change following five hours induction of U937-PR9 cells (this study) and U937-PR9 cells induced for varying times (4, 6, 12 and 24 hours) (Wang et al 2020.). Each box plot has an accompanying bar plot that reports the percentage of genes that have expression levels greater than a 0.5 log2 fold change when compared to their matched uninduced cells. N = 1 RNA-seq experiment per condition taken from Wang et al 2020. **s** Notched box plots showing the H3K9/K14ac read coverage at gene promoters. Light blue = upregulated in uninduced U937-PR9 cells, cyan= upregulated in induced U937-PR9 cells, pink = downregulated in uninduced U937-PR9 cells, red= downregulated in induced U937-PR9 cells. * indicates the two-sided Wilcoxon rank test p-value when comparing the normalized H3K9/K14ac read counts at upregulated genes in uninduced vs induced cells. N = 2 biological replicates of each ChIP-seq condition. **t** Notched box plots showing the PML::RARA binding strength (based on the SEACR peak calling score) of peaks at upregulated gene promoters (green),

downregulated gene promoters (red) and promoters with no change in expression (grey). * indicates the two-sided t-test p-value for each comparison. N = 2 biological replicates of each Cut&Run condition. **u** Notched box plots showing the width of PML::RARA peaks at upregulated gene promoters (green), downregulated gene promoters (red) and promoters with no change in expression (grey). * indicates the two-tailed t-test p-value for each comparison. N = 2 biological replicates of each Cut&Run condition. **v** Notched box plot showing the absolute log-fold change (vs uninduced) distributions of significantly upregulated genes and downregulated genes. * indicates the two-sided t-test p-value for each comparison. N = 2 biological replicates of each RNA-seq condition. All boxplots were plotted with identical parameters: minima and maxima are indicated either as the lowest and highest outliers or as the lower and upper whiskers if the values are within -1.5 and 1.5x the interquartile range from the lower and upper quartiles, respectively. The box bounds correspond to the first and third quartiles. The center indicates the median - all outliers are plotted. Source data are provided as a Source Data file – the corresponding data are also used in the main Figure 2.

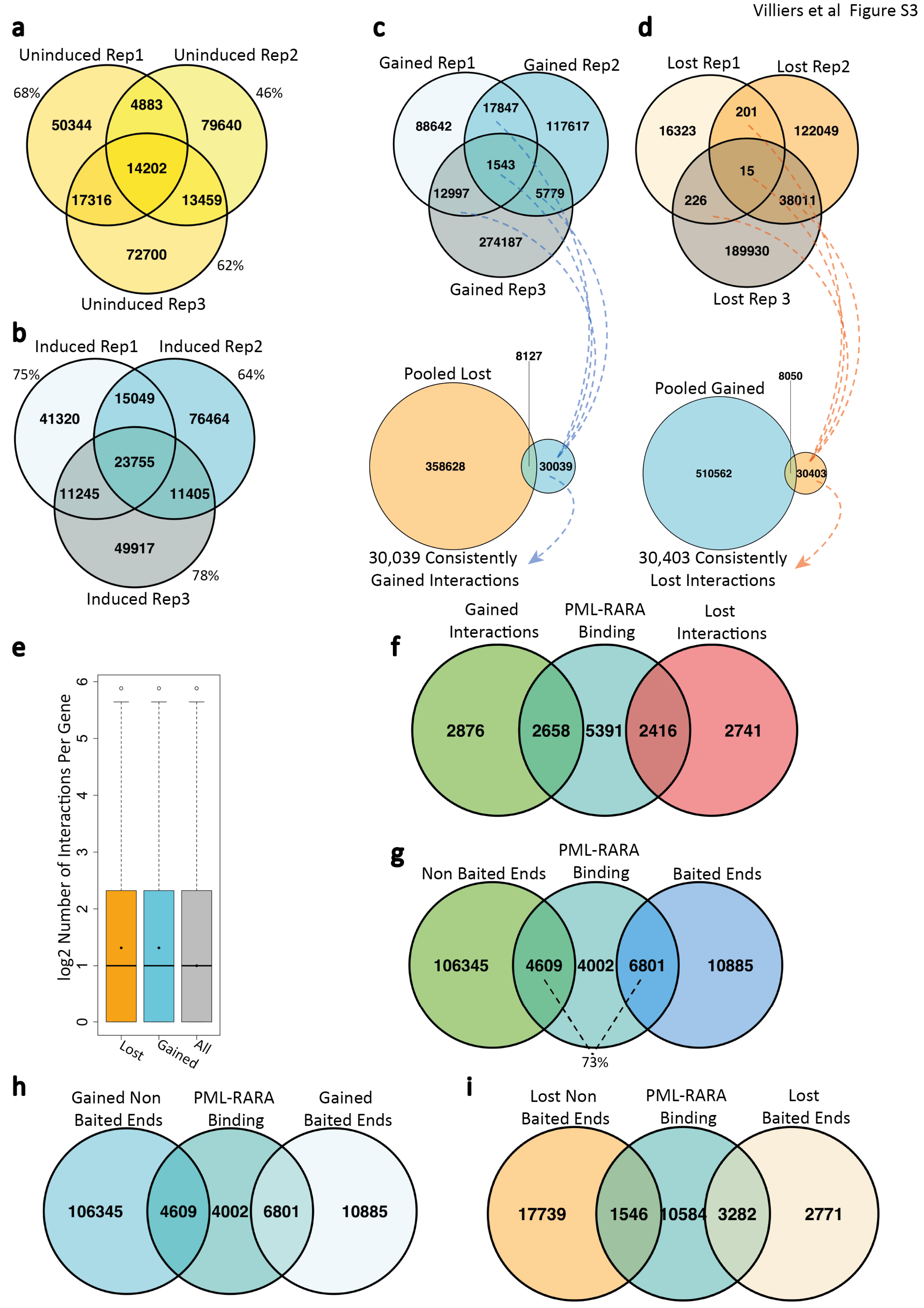

Supplementary Fig. 3: CHi-C + differential interactions.

a Venn diagram showing the overlap of significant interactions called by the CHiCAGO interaction-calling pipeline in each capture Hi-C replicate experiment in uninduced U937-PR9 cells. The

percentage of interactions that overlap at least one other replicate is shown. **b** Venn diagram showing the overlap of significant interactions called by the CHiCAGO interaction-calling pipeline in each capture Hi-C replicate experiment in induced U937-PR9 cells. The percentage of interactions that overlap at least one other replicate is shown. **c** Venn diagrams displaying identification of consensus significantly gained interactions, which included significant interactions that were present in at least two replicates (above). The list of gained interactions excluded interactions that were lost in any of the three experiments. **d** Venn diagrams displaying identification of consensus significantly lost interactions, which included significant interactions that were present in at least two replicates (above). The list of lost interactions excluded interactions that were gained in any of the three experiments (bottom). **e** Box plot showing the distribution of differential interaction numbers per gene from the 60,442 differential interactions detected. Y-axis is in log2 scale. Orange box shows the numbers of lost interactions per gene. Cyan shows the numbers of gained interactions per gene. Grey box shows the numbers of all differential interactions per gene. Black dot indicates the mean number of lost/gained interactions. N = 3 biological replicates of each capture Hi-C condition. **f** Venn diagram showing the overlap of all genes with lost interactions (red), genes with gained interactions (light green) and genes with PML::RARA binding (dark green). **g** Venn diagram showing the overlap of the baited ends (blue) and non-baited ends (green) of all significant interactions with PML::RARA binding sites (dark green). **h** Venn diagram showing the overlap of gained baited ends (cyan) and gained non-baited ends (white) of significant gained interactions with PML::RARA binding sites (dark green). **i** Venn diagram showing the overlap of the baited ends (orange) and non-baited ends (light orange) of significant lost interactions with PML::RARA binding sites (dark green). . All boxplots were plotted with identical parameters: minima and maxima are indicated either as the lowest and highest outliers or as the lower and upper whiskers if the values are within -1.5 and 1.5x the interquartile range from the lower and upper quartiles, respectively. The box bounds correspond to the first and third quartiles. The center indicates the median - all outliers are plotted. Source data are provided as a Source Data file – the corresponding data are also used in the main Figure 3.

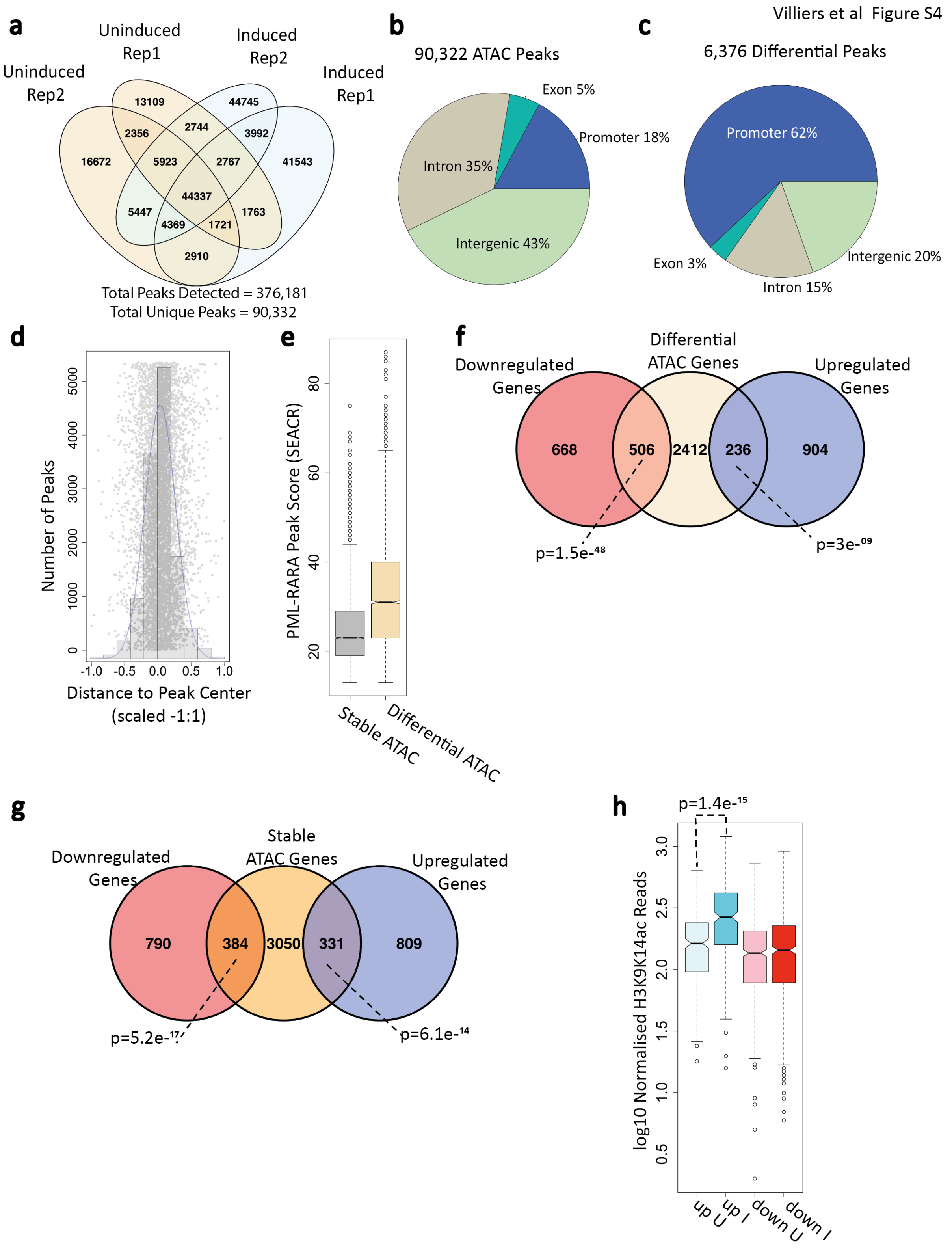

Supplementary Fig. 4: Chromatin accessibility elucidation.

a Venn diagram showing the overlap of unique ATAC-seq peaks in uninduced and induced replicates of U937-PR9 cells (q-value <= 0.01). **b** Pie chart showing the distributions across genomic features of the 90,322 reproducible ATAC-seq peaks. **c** Pie chart showing the distributions across genomic features of the 6,376 differential ATAC-seq peaks. **d** Histogram showing the position frequency of PML::RARA binding sites relative to the center of ATAC-seq peaks. ATAC-seq peaks were scaled to span

from –1 to 1 with 0 being the center of the peak. **e** Notched box plots showing the PML::RARA binding score (based on the SEACR peak score) of PML::RARA peaks overlapping with non-differential (stable) ATAC-seq peaks (grey) and differential ATAC-seq peaks (orange). N = 2 biological replicates of each Cut&Run condition. **f** Venn diagram showing overlaps of genes with differential ATAC-seq peaks at their promoters (orange) and differentially expressed genes (upregulated = blue, downregulated = red). The significance of each overlap is represented as the p-value using a chi-squared test. **g** Venn diagram showing overlaps of genes with stable ATAC-seq peaks at their promoters (orange) and differentially expressed genes (upregulated = blue, downregulated = red). The significance of each overlap is represented as the p-value using a chi-squared test. **h** Notched box plots showing the H3K9/K14ac read coverage at up and downregulated ATAC-seq peaks, associated with differentially expressed genes. Light blue = upregulated in uninduced (U) U937-PR9 cells, cyan= upregulated in induced (I) U937-PR9 cells, pink = downregulated in uninduced (U) U937-PR9 cells, red= downregulated in induced (I) U937-PR9 cells. * indicates the two-sided Wilcoxon rank test p-value when comparing the H3K9/K14ac read build up at upregulated gene in the uninduced U937-PR9 cells vs upregulated genes in induced U937-PR9 cells. N = 2 biological replicates of each ChIP-seq condition. All boxplots were plotted with identical parameters: minima and maxima are indicated either as the lowest and highest outliers or as the lower and upper whiskers if the values are within -1.5 and 1.5x the interquartile range from the lower and upper quartiles, respectively. The box bounds correspond to the first and third quartiles. The centre indicates the median - all outliers are plotted. Source data are provided as a Source Data file – the corresponding data are also used in the main Figure 4.

Villiers et al Figure S5

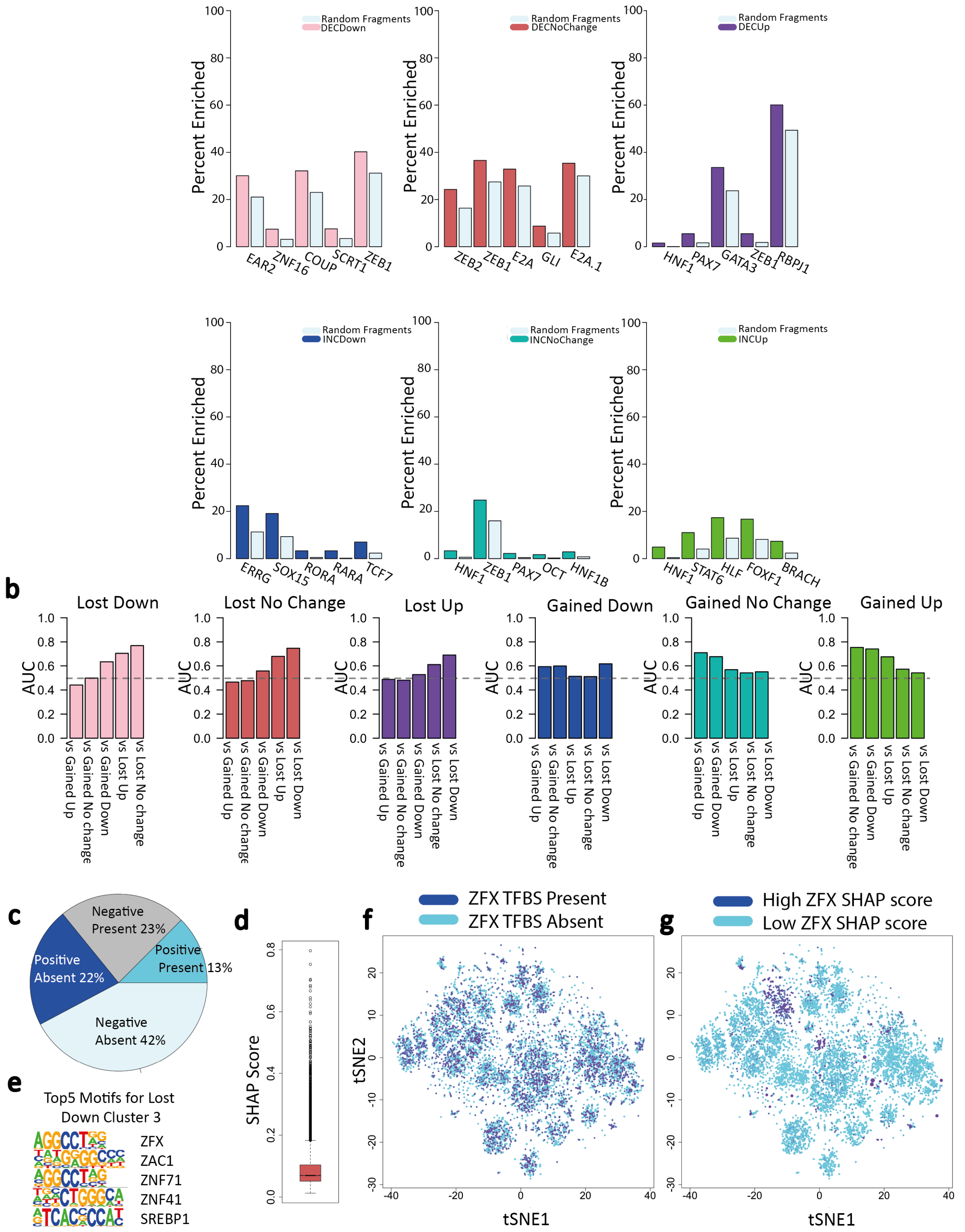

Supplementary Fig. 5: Machine learning.

a Bar plots showing the percent enrichment of the top five most significantly enriched motifs in each fragment category. Each motif has two bars, one being the % enrichment in the category, and the second (light cyan) is the % enrichment in the background fragments (pink, lost/downregulated; brown, lost/no change; purple, lost/upregulated; blue, gained/downregulated; teal, gained/no change; green, gained/upregulated). **b** Bar plots showing the Area Under the Curve scores for each

one-vs-one binary classification. Each plot represents one category vs all other categories in a pairwise comparison. **c** Pie chart showing the % of the top 15 SHAPELY drivers and their influences, based on whether the TFBS is present or absent. **d** Boxplot showing the distribution of the top SHAPELY weighting for correctly predicted fragments across all 6 fragment categories. **e** Motifs for top five TFBS that drive the prediction for the downregulated/lost interaction cluster #3. **f** tSNE as described in Fig. 5d, here, black dots represent fragments with at least one ZFX binding site. **g** tSNE as described in Fig. 5d, here, the size of the black dots represents the SHAPELY weighting for the ZFX motif. Larger dots = higher positive score. All boxplots were plotted with identical parameters: minima and maxima are indicated either as the lowest and highest outliers or as the lower and upper whiskers if the values are within -1.5 and 1.5x the interquartile range from the lower and upper quartiles, respectively. The box bounds correspond to the first and third quartiles. The center indicates the median - all outliers are plotted. Source data are provided as a Source Data file – the corresponding data are also used in the main Figure 5.

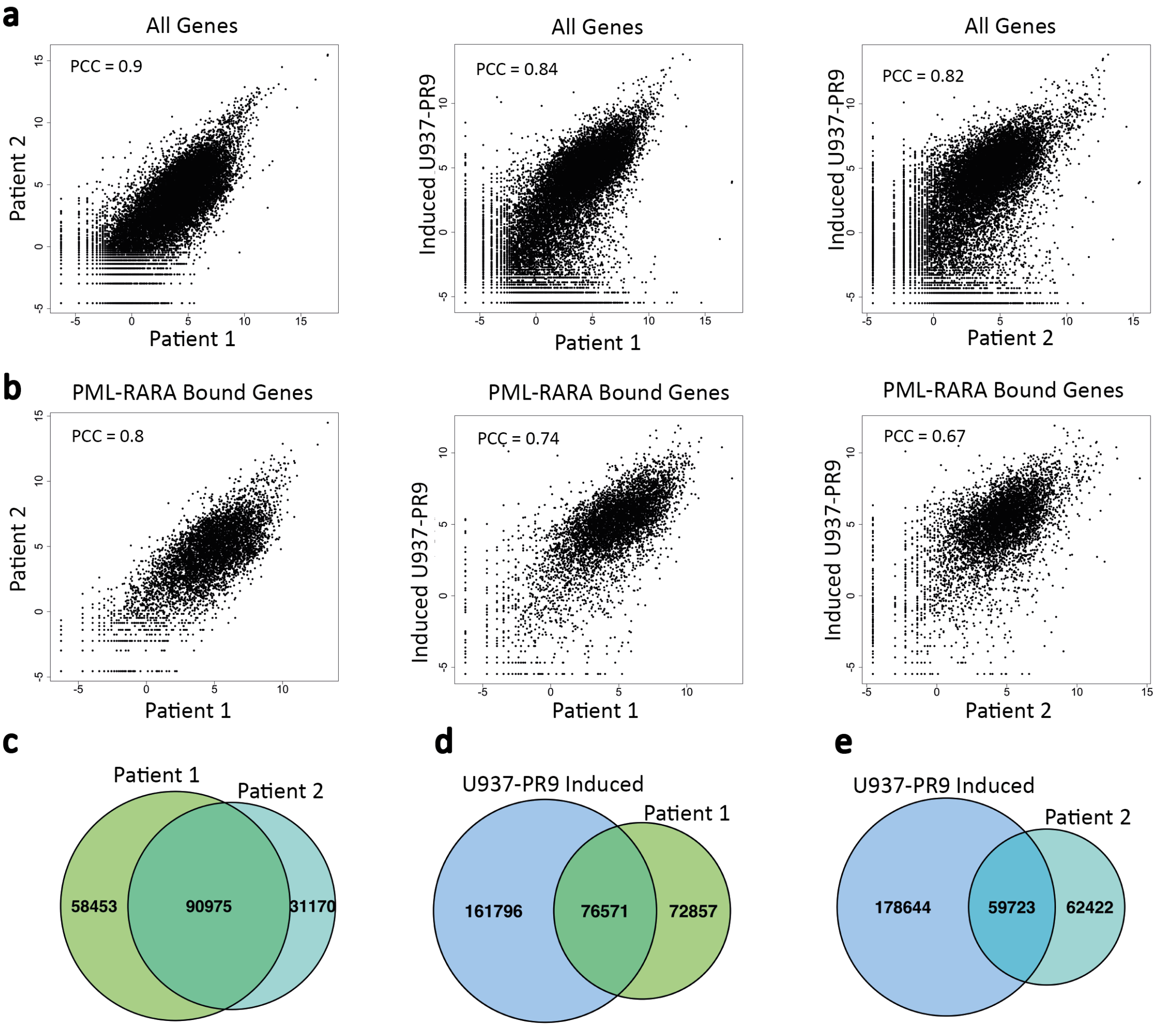
a
All Genes
PCC = 0.9
Patient 2
Patient 1
All Genes
PCC = 0.84
Induced U937-PR9
Patient 1
All Genes
PCC = 0.82
Induced U937-PR9
Patient 2
b
PML-RARA Bound Genes
PCC = 0.8
Patient 2
Patient 1
PML-RARA Bound Genes
PCC = 0.74
Induced U937-PR9
Patient 1
PML-RARA Bound Genes
PCC = 0.67
Induced U937-PR9
Patient 2
c
Patient 1
Patient 2
58453
90975
31170
d
U937-PR9 Induced
Patient 1
161796
76571
72857
e
U937-PR9 Induced
Patient 2
178644
59723
62422

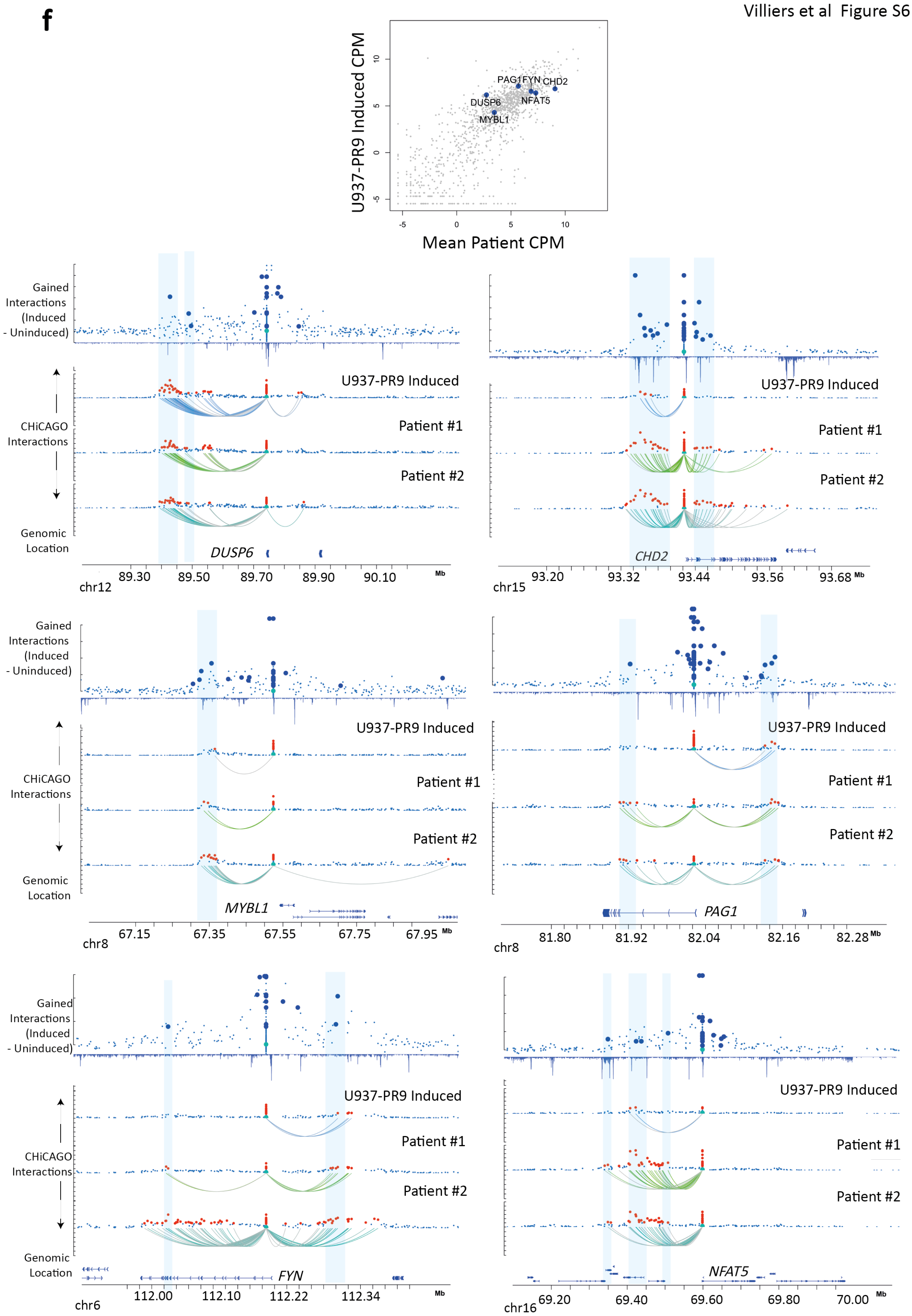
f
U937-PR9 Induced CPM
Mean Patient CPM
PAG1
FYN
CHD2
DUSP6
NFAT5
MYBL1
Gained Interactions (Induced - Uninduced)
CHiCAGO Interactions
Genomic Location
U937-PR9 Induced
Patient #1
Patient #2
DUSP6
chr12
89.30
89.50
89.70
89.90
90.10
Mb
CHD2
chr15
93.20
93.32
93.44
93.56
93.68
MYBL1
chr8
67.15
67.35
67.55
67.75
67.95
PAG1
chr8
81.80
81.92
82.04
82.16
82.28
FYN
chr6
112.00
112.10
112.22
112.34
NFAT5
chr16
69.20
69.40
69.60
69.80
70.00

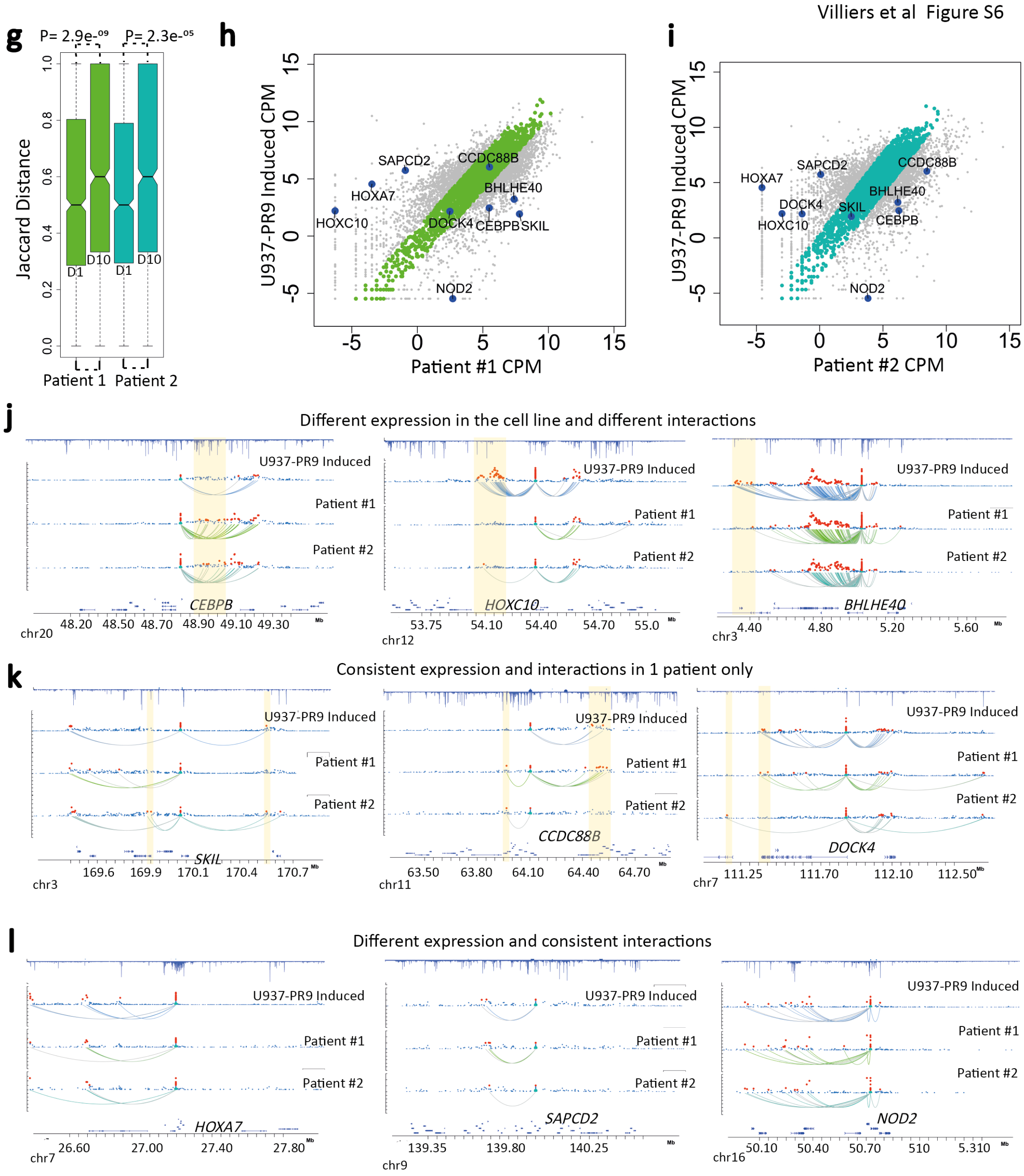

Supplementary Fig. 6: Gene expression and long-range interactions in APL patients.

a Scatter plots showing the correlation of normalized CPM from RNA-seq experiments comparing patients and the U937-PR induced cells. Left plot shows Patient #1 (x-axis) vs Patient #2 (y-axis). Center plot shows Patient #1 (x-axis) vs induced U937-PR9 (y-axis). Right plot shows Patient #2 (x-axis) vs induced U937-PR9 (y-axis). PCC = Pearson Correlation Coefficient. **b** Scatter plots showing the correlation of PML::RARA bound genes. Left plot shows Patient #1 (x-axis) vs Patient #2 (y-axis). Centre plot shows Patient #1 (x-axis) vs induced U937-PR9 (y-axis). Right plot shows Patient #2 (x-axis) vs induced U937-PR9 (y-axis). PCC = Pearson Correlation Coefficient. **c** Venn diagram showing the overlap of the significant interactions in Patient #1 (light green) and Patient #2 (dark green). **d** Venn diagram showing the overlap of the significant interactions in the induced U937-PR9 cells (blue) and Patient #1 (light green). **e** Venn diagram showing the overlap of significant interactions in the induced U937-PR9

cells (blue) and Patient #2 (dark green). **f** The top scatter plot shows the mean expression correlation of the 1,357 genes, comparing patients and induced U937-PR9 cells. Pearson correlation coefficient = 0.87. Positions of six genes relating to the landscape plots below are shown (blue dots). Interaction landscape plots of six genes: *DUSP6*, *CHD2*, *MYBL1, PAG1*, *FYN and NFAT5*. The top panel in each plot shows the gained interactions after PML::RARA induction in the U937-PR9 cells, large blue dots indicate regions with a significant differential (gained) interaction, as determined by GOTHiC (ihw <= 0.01). The blue inverted peak tracks show the binding profiles of PML::RARA in U937-PR9 cells. Arc plots show the interactions for the induced U937-PR9 cells (blue), Patient#1 (light green) and Patient#2 (dark green). The colored arcs connect significant interactions to the gene promoter. Significant interactions are displayed as a red dot, and non-significant interactions as a blue dot. Each dot represents a unique HindIII fragment. **g** Notched box plots showing the similarity of interaction profiles between genes with the most and least similar expression output. Interaction similarity is measured by Jaccard Distance, where the smaller the distance the greater the similarity. P indicates the two-sided t-test p-value comparing the similarity scores for genes in the top and bottom 10th percentiles of expression similarity. Patient #1 profiles are represented by light green and Patient #2 profiles are represented by dark green. N = 1 capture Hi-C library generated per patient sample each box represents ~2,000 genes. **h** Plot showing the gene expression correlation of PML::RARA bound genes for Patient #1 and induced U937-PR9 cells. Black dots show nine genes with varying expression similarities. The green coloring shows genes within the top two quartiles of expression similarity, based on R^2. The interaction profiles of these genes are interrogated in Supplementary fig. 6j-l. **i** Plot showing the gene expression correlation of PML::RARA bound genes for Patient #2 and induced U937-PR9 cells. Black dots show nine genes with varying expression similarities with the cell line. The dark green coloring shows genes within the top two quartiles of expression similarity based on R^2. The interaction profiles of these genes are interrogated in Supplementary fig. 6j-l. **j** Interaction landscape plots of three genes that have different expression levels and interaction profiles in both patients when compared to induced U937-PR9 cells: *CEBPB, HOXC10* and *BHLHE40*. The inverted blue peak tracks show the read coverage of PML::RARA binding in induced U937-PR9. Each plot also contains the interactions for the induced U937-PR9 (blue arcs), Patient#1 (light green arcs) and Patient#2 (dark green arcs). The colored arcs connect significant interactions to the gene promoter, a significant interaction is a red dot, a nonsignificant interaction is a blue dot. Each dot represents a unique HindIII fragment. **k** Interaction landscape plots of three genes that have consistent expression levels and interaction profiles in only one patient sample when compared to induced U937-PR9 cells: *SKIL* is consistent in Patient #2, *CCDC88B* and *DOCK4* are consistent in Patient #1. The inverted blue peak tracks show the read coverage of PML::RARA binding in induced U937-PR9. Each plot also contains the interactions for the induced U937-PR9 (blue arcs), Patient #1 (light green arcs) and Patient #2 (dark green arcs). The colored arcs connect significant interactions to the gene promoter, a significant interaction is a red dot, a nonsignificant interaction is a blue dot. Each dot represents a unique HindIII fragment. **l** Interaction landscape plots of three genes that have different expression levels but consistent interaction profiles in both patients and induced U937-PR9 cells: *NOD2*, *HOXA7* and *SAPDC2*. The inverted blue peak tracks show the read coverage of PML::RARA binding in induced U937-PR9. Each plot also contains the interactions for the induced U937-PR9 (blue), Patient #1 (light green arcs) and Patient #2 (dark green arcs). The colored arcs connect significant interactions to the gene promoter, a significant interaction is a red dot, a nonsignificant interaction is a blue dot. Each dot represents a unique HindIII fragment. All boxplots were plotted with identical parameters: minima and maxima are indicated either as the lowest and highest outliers or as the lower and upper whiskers

if the values are within -1.5 and 1.5x the interquartile range from the lower and upper quartiles, respectively. The box bounds correspond to the first and third quartiles. The center indicates the median - all outliers are plotted. Source data are provided as a Source Data file – the corresponding data are also used in the main Figure 6.

Supplementary Table 1 with statistical test descriptions of all frequentist inferential statistics from all figures			
**FIGURE**	**Statistical test**	**Statistics**	**Boxplot Statistics**
**Figure 2c**	Chi-squared	DOWNREGULATED - X-squared = 70.3, df = 1, p-value = 1.9e-14 | UPREGULATED - X-squared = 0.04, df = 1, p-value = 0.007	
**Figure S2i**	t-test (two-sided)	t = -65.438, df = 92,1,p-value = 4.5e-79, 95CI = -18.73, -17.63	(min, first quartile, median, third quartile, max) - Uninduced peaks: 8, 8, 8, 9, 10. Induced peaks: 13, 19, 25, 33, 54.
**Figure S2l**	Chi-squared	X-squared = 16341, df = 1, p-value = 2.2e-100	
**Figure S2m**	t-test (two-sided)	t = 12.62, df = 14750, p-value = 2.4e-36, 95CI = 1.802, 2.465	(min, first quartile, median, third quartile, max) - -Promoter peaks: 13, 20, 26, 35, 57. Non-promoter peaks: 14, 19, 24, 31, 49
**Figure S2n**	t-test (two-sided)	t = 43.12, df = 13777, p-value < 3.6e-43	(min, first quartile, median, third quartile, max) - Promoter peaks: 77, 757, 1096, 2528, 2657. Non-promoter peaks: 79, 440, 637, 951, 1717.
**Figure S2s**	Wilcox test (two-sided)	W = 1121912, p-value = 1.4e-47	(min, first quartile, median, third quartile, max) - U: 0.47, 1.41, 1.75, 2.04, 2.83. I: 0.77, 1.63,1.98,2.26, 3.01. U: 0.77, 1.56, 1.86, 2.12, 2.85. I: 0.77, 1.59, 1.91, 2.17, 2.85.
**Figure S2t**	t-test (two-sided)	UPREGULATED - t = 10.5, df = 583.9, p-value = 9.2e-24, 95CI = 234.3, 342.1 | DOWNREGULATED - t = 17.11, df = 889.8, p-value = 1.0e-56, 95CI = 470.87, 592.86 | UPvsDOWNREGULATED - t = -5.97, df = 1373, p-value = 12.98e-09, 95CI = -323.69, -163.62	(min, first quartile, median, third quartile, max) - Up genes: 13, 21, 26, 34, 52. No change genes: 13, 19, 24, 33, 54. Down genes: 15, 23, 31, 39, 63.
**Figure S2u**	t-test (two-sided)	UPREGULATED - t = 1.81, df = 589, p-value = 0.07, 95CI = -0.06, 1.64 | DOWNREGULATED - t = 11.255, df = 924, p-value = 1.9e-27 | UPvsDOWNREGULATED - t = -6.5, df = 1275, p-value = 1.08e-10, 95CI = 3.78, 5.38	(min, first quartile, median, third quartile, max) - Up genes: 101, 808, 1107.5, 1561, 2615. No change genes: 77, 516, 795, 1216, 2265. Down genes: 136, 887, 1323, 1841, 3266.
**Figure S2v**	t-test (two-sided)	t = 1.6, df. 921, p-value = 0.11, 95CI = -0.0093, 0.918	(min, first quartile, median, third quartile, max) - Up genes: 0.25, 0.38, 0.50, 0.70, 1.17. Down genes: 0.24, 0.36, 0.48, 0.71, 1.23.
**Figure 3b**	Boxplot	n/a	(min, first quartile, median, third quartile, max) - uninduced: 0, 1.58, 2.8, 3.8, 7.13. Induced: 0, 1.58, 2.8, 3.9, 7.3.
**Figure 3c**	Boxplot	n/a	
**Figure 3e**	Chi-squared	DOWNREGULATED - X-squared = 0.86, df = 1, p-value = 0.86 | UPREGULATED - X-squared = 62.5, df = 1, p-value = 2.6e-15	
**Figure 3c**	Boxplot	n/a	(min, first quartile, median, third quartile, max) - uninduced Q1: 0, 1.58, 2.58, 3.70, 6.84. uninduced Q2: 0, 1.58, 2.81, 3.81, 7.12. uninduced Q3: 0, 2, 3.17, 4.08, 7.19. uninduced Q4: 0, 2, 3.32, 4.32, 7.74. induced Q1: 0, 1.54, 2.5, 3.68, 6.9. induced Q2: 0, 1.54, 2.5, 3.78, 6.9. induced Q3: 0, 2, 3.32, 4.32, 7.86. uninduced Q4: 0, 2.45, 3.55, 4.42, 7.76.
**Figure 3f**	Chi-squared	DOWNREGULATED - X-squared =52.1, df = 1, p-value = 5.2e-13 | UPREGULATED - X-squared = 1.61, df = 1, p-value = 0.2	
**Figure 3g**	t-test (two-sided)	GAINING - t = 8.77, df - 1648, p-value = 4.1e-18, 95CI = 0.0970, 0.1528 | LOSING - t = -2.38, df = 1490, p-value = 0.0017, 95CI = -0.0601, -0.0059	(min, first quartile, median, third quartile, max) - Gaining interactions: -0.63, -0.11, 0.05, 0.25, 0.77. Both: -0.62, -0.17, -0.03, 0.13 , 0.59. Losing Interactions: -0.76, -0.22, -0.06, 0.13 , 0.66.
**Figure S3e**	Boxplot	n/a	(min, first quartile, median, third quartile, max) - Lost: 0, 0, 1, 2.25, 5.95. Gained: 0, 0, 1, 2.25, 5.95. All: 0, 0, 1, 2.25, 5.95.
**Figure S4e**	Boxplot	n/a	(min, first quartile, median, third quartile, max) - Stable ATAC: 13, 19, 24, 31, 49. Differential ATAC: 15, 23, 31, 41, 68.
**Figure S4f**	Chi-squared	DOWNREGULATED - X-squared = 239.35, df = 1, p-value = 1.5e-48 | UPREGULATED - X-squared = 34.1, df = 1, p-value =3e-09	
**Figure S4g**	Chi-squared	DOWNREGULATED - X-squared = 87.42, df = 1, p-value = 5.2e-17 | UPREGULATED - X-squared = 56.32, df = 1, p-value =6.1e-14	
**Figure S4h**	Wilcox test (two-sided)	W = 17172, p-value = 1.473e-15	(min, first quartile, median, third quartile, max) Up U: 1.51, 1.98, 2.11, 2.31, 2.68. Up I: 1.66, 2.18, 2.34, 2.55, 2.94. Down U: 1.25, 1.80, 2.02, 2.24, 2.69. Down I: 1.03, 1.76, 2.06, 2.29, 2.82.
**Figure S5d**	Boxplot	n/a	(min, first quartile, median, third quartile, max) - 0, 0.07, 0.08, 0.095, 0.8
**Figure S6g**	t-test (two-sided)	PATIENT1 - p-value = 2.9e-9, PATIENT2 - p-value = 2.3e-5	(min, first quartile, median, third quartile, max) - Patient1 D1: 0, 0.29, 0.49, 0.8, 1. Patient #1 D10: 0, 0.36, 0.6, 1, 1. Patient #2 D1: 0, 0.30, 0.49, 0.78, 1. Patient2 D10: 0, 0.36, 0.6, 1, 1.

a) Figure provided in Supplementary Figure 1a

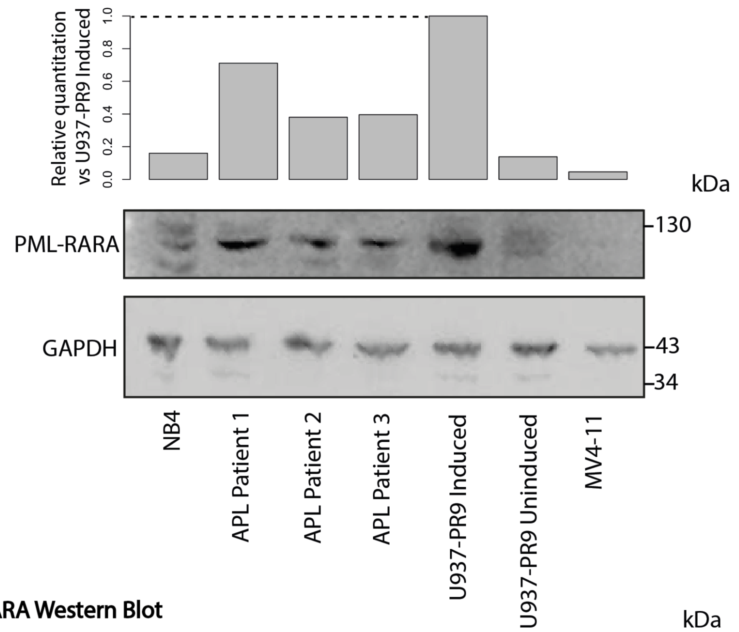

b) Uncropped RARA Western Blot

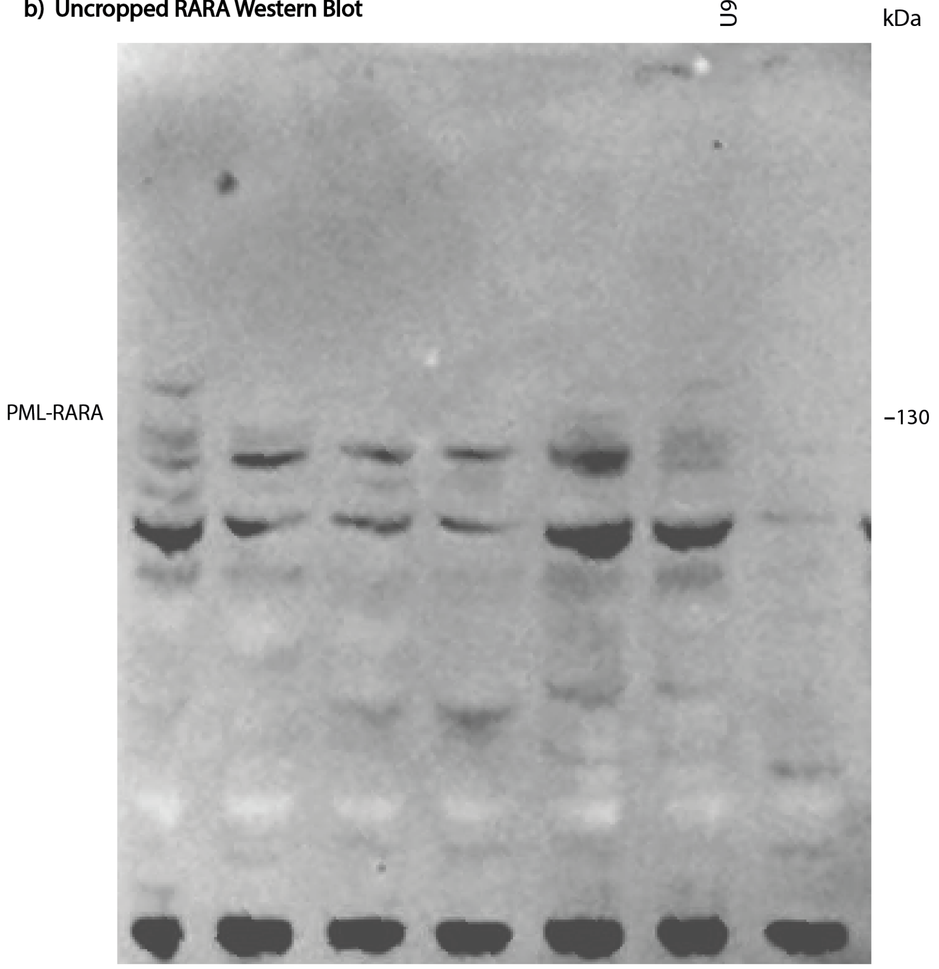

c) Uncropped GAPDH Western Blot

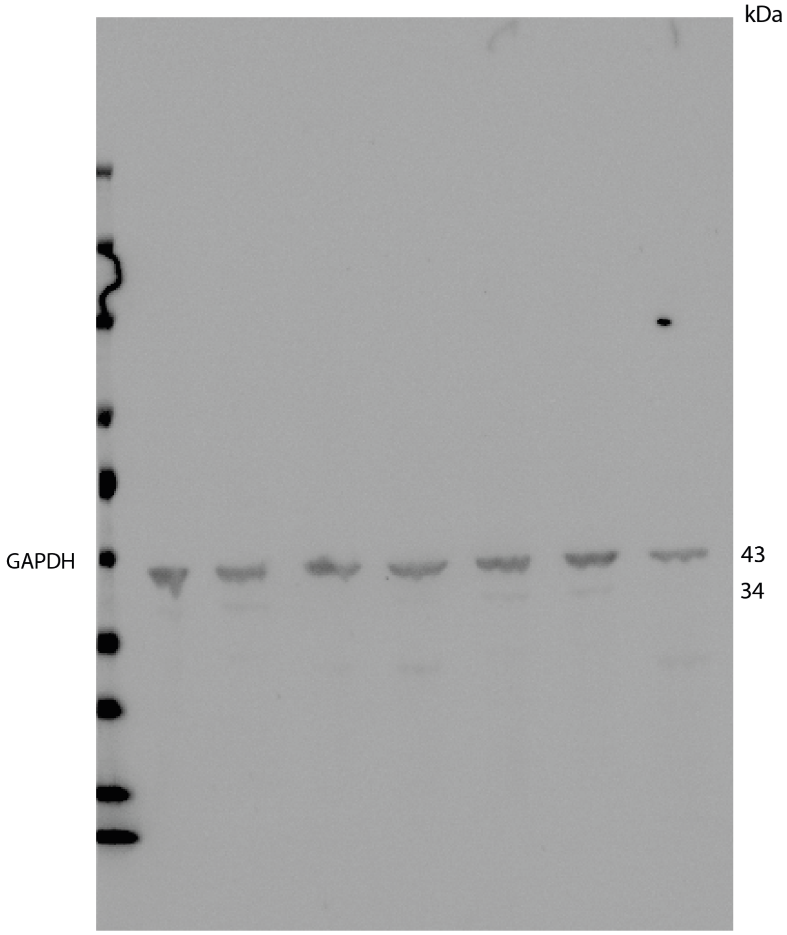

Supplement: Supplementary file 1 — Supplementary information [file 41467_2023_36262_MOESM1_ESM.pdf]
